# Comprehensive Assessment of the Virulence Factors *sub 3*, *sub 6* and *mcpA* in the Zoonotic Dermatophyte *Trichophyton benhamiae* Using FISH and qPCR

**DOI:** 10.3390/jof8010024

**Published:** 2021-12-28

**Authors:** Christina-Marie Baumbach, Antje Rückner, Lena Partusch, Eric Engel, Wieland Schrödl, Jule Kristin Michler

**Affiliations:** 1 Centre of Infectious Diseases, Faculty of Veterinary Medicine, Institute of Bacteriology and Mycology, Leipzig University, An den Tierkliniken 29, D-04103 Leipzig, Germany; christina-marie-baumbach@vetmed.uni-leipzig.de (C.-M.B.); schroedl@vetmed.uni-leipzig.de (W.S.); 2 Centre of Infectious Diseases, Faculty of Veterinary Medicine, Institute of Virology, Leipzig University, An den Tierkliniken 29, D-04103 Leipzig, Germany; antje.rueckner@sanktgeorg.de (A.R.); Eric.engel@web.de (E.E.); 3 Faculty of Veterinary Medicine, Institute of Veterinary Anatomy, Histology and Embryology, Leipzig University, An den Tierkliniken 43, D-04103 Leipzig, Germany; lp42jewo@studserv.uni-leipzig.de

**Keywords:** *Trichophyton benhamiae*, subtilisin, metallocarboxypeptidase, virulence factors, qPCR, in situ hybridization, guinea pig skin explants, FISH

## Abstract

Skin infections by keratinophilic fungi are commonly referred to as dermatophytosis and represent a major health burden worldwide. Although patient numbers are on the rise, data on virulence factors, their function and kinetics are scarce. We employed an ex vivo infection model based on guinea pig skin explants (GPSE) for the zoonotic dermatophyte *Trichophyton* *(T.) benhamiae* to investigate kinetics of the virulence factors subtilisin *(sub) 3*, *sub 6*, *metallocarboxypeptidase A (mcpA)* and *isocitrate lyase (isol)* at gene level for ten days. Fluorescence in situ hybridization (FISH) and quantitative polymerase chain reaction (qPCR) were used to detect and quantify the transcripts, respectively. Kingdom-spanning, species-specific and virulence factor-specific probes were successfully applied to isolated fungal elements showing inhomogeneous fluorescence signals along hyphae. Staining results for inoculated GPSE remained inconsistent despite thorough optimization. qPCR revealed a significant increase of *sub 3*- and *mcpA*-transcripts toward the end of culture, *sub 6* and *isol* remained at a low level throughout the entire culture period. Sub 3 is tightly connected to the de novo formation of conidia during culture. Since *sub 6* is considered an in vivo disease marker. However, the presented findings urgently call for further research on the role of certain virulence factors during infection and disease.

## 1. Introduction

Dermatophytoses, i.e., superficial fungal infections of skin and its appendages, are diagnosed in communities with a low socioeconomic status but also in urbanized regions with modern habits and diseases of civilization. Concisely, distribution, etiological agent and clinical manifestation may vary with geographical localization and economic and cultural factors, but dermatophytoses are a global threat to human health [1]. Furthermore, the growing number of susceptible hosts—even among immunocompetent individuals—and reports of antifungal resistances in many dermatophyte species [2,3] highlight the need for a better understanding of host-pathogen interactions and the molecular biological mechanisms underlying infection and disease. With the growing understanding that artificial in vitro systems cannot depict the in vivo situation adequately [4,5,6] and, concomitantly, that animal experiments need to be reduced to an absolute minimum, ex vivo models are considered attractive alternative experimental approaches to explore the aforementioned open questions [7,8].

For the zoonotic dermatophyte *Trichophyton (T.) benhamiae*, such an ex vivo model based on guinea pig skin explants (GPSE) was previously established by us [9]. Briefly, standardized inocula of the dermatophytes’ conidia were applied to GPSE in a transwell cell culture system. Adhesion, invasion and infection of the skin explants by fungal elements were monitored for up to ten days and the important virulence factors subtilisin (Sub) 3 and 6 as well as metallocarboxypeptidase A (MCPA) were assessed at protein level using immunofluorescence (IF) analyses [10]. 

Secreted proteolytic enzymes are the most studied virulence factors in dermatophytes [11,12] but they are still controversially discussed in terms of distinct function and general occurrence. Sub 3 protease seems to play an essential role for conidial adhesion to epidermal structures of different hosts [13,14]; Sub 6 is considered the major in vivo disease marker [5,6,15]. However, sometimes even contradictory reports using different experimental set ups and analysis methods impede an unequivocal assignment to certain laboratory conditions (in vitro vs. in vivo) and/or a clinical status, let alone the definition of its significance in each context [4]. This warrants further research, especially in terms of quantitative data.

Fluorescence in situ hybridization (FISH) is a well-documented method in research and diagnostics to visualize nucleic acid targets in their cellular environment [16]. The detection and identification of microorganisms in general and of clinically relevant fungi in particular using FISH in different kinds of specimens are often described, e. g. *Candida* sp. in blood cultures [17] and *Cryptococcus neoformans* in cerebrospinal fluid [18]. Furthermore, *Aspergillus, Fusarium, Rhizopus* and other mold species were found in formalin-fixed paraffin-embedded (FFPE) tissue sections, even in mixed fungal infections [19,20,21]. However, to the best of our knowledge, there are no reports of a FISH-based detection of dermatophytes and/or their virulence factors in skin or its appendages.

Quantitative or real-time polymerase chain reaction (qPCR) has become one of the standard techniques in life sciences and molecular diagnostics since it allows for the detection, quantification and further characterization of minute amounts of nucleic acid targets in a variety of samples [22,23]. Yet, there is a lack of quantitative data on the dermatophytes’ mode of life including virulence factors obtained with this speedy and sensitive methodology. There are only a few pioneer studies basically proving the transcription of fungalysins (i.e., metalloproteases *mep* 1–5) and subtilisins during host infection [5,24,25]. However, these were proof-of-principle studies using a limited number of samples for conventional PCR and relative qPCR.

To start filling this knowledge gap on virulence factor production at gene level, we employed FISH to qualitatively detect *sub 3*, *sub 6* and *mcpA* and used qPCR in the abovementioned ex vivo model to generate comparable quantitative data.

## 2. Materials and Methods

### 2.1. Fungal Isolates, GPSE Culture and Infection Experiments

Dermatophyte isolates were recovered from human patients (*n* = 10; obtained from the Laboratory of Medical Microbiology, Mölbis; with informed patient consent) and infected Guinea pigs (Gp; *n* = 10, samples derived from feed animals at a local zoological garden). After 7 d of growth on Sabouraud–Dextrose agar (4%, 28 °C), species identity was confirmed morphologically (see Supplementary Table S1) and microbiologically. For the latter, fungal DNA was isolated using the QIAamp® DNA Mini Kit (cat. no. 51304, Qiagen, Hilden, Germany) with an additional overnight Proteinase K digestion at 56 °C and 600 rpm agitation. The *internal transcribed spacer* (ITS) region was amplified using the universal primers LSU266 and V9G [26] (10 µmol, 1µL each; synthesized by Microsynth Seqlab, Goettingen, Germany) and the Red HS Taq Master Mix (2×; cat. co. 331126S, Biozym, Oldendorf, Germany) with the following thermal profile: after preheating for 5 min at 95 °C, 30 cycles of denaturation for 1 min at 95 °C, annealing for 1 min at 55 °C and extension for 1 min at 72 °C followed. A final extension of 1 min at 72 °C concluded each run [26]. PCR products were purified using the QIAquick® PCR Purification Kit (cat. no. 28104, Qiagen), Sanger sequencing was performed by Microsynth Seqlab. Dermatophytes were identified and ITS-typified by similarity search using the Basic Local Alignment Search Tool (BLASTn; https://blast.ncbi.nlm.nih.gov/Blast, accessed on 1 December 2021). For further characterization, the mating type of the isolates was determined. Again, the Red HS Taq Master Mix was used together with primers MF1 and MF2 or MF3 and MF4 [27] (10 µmol, 1 µL each), respectively, in two separate PCRs for each dermatophyte isolate (1 µL genomic DNA). The reaction mixture (total volume of 20 µL) was incubated for 1 min at 95 °C, subjected to 40 cycles of 15 s at 95 °C and 15 s at 72 °C, and finally incubated for 10 min at 72 °C. All primers used during this study are listed in Supplementary Table S2.

Fungal inocula consisted of 1 × 10^3^ conidia dissolved in 2 µL phosphate buffered saline (PBS) derived from the above-mentioned *T. benhamiae* isolates subjected to culture conditions favoring conidia production [10].

GPSE culture and infection experiments were carried out essentially as described previously [9,10]. Briefly, skin explants were prepared from clipped and disinfected flank regions of Gp euthanized for reasons not related to this study and according to local ethical guidelines and state law. Skin explants (approximately 2mm × 2 mm) were placed in a transwell cell culture system, provided with standard culture media (supplemented with Gp serum and growth factors [9]) and directly inoculated with the conidial suspensions [10]. GPSE were incubated at 30 °C, 5% CO_2_ and 95% relative humidity; samples were taken after 3, 5, 7 and 10 d of culture and formalin-fixed and paraffin embedded (FFPE) according to standard protocols or frozen at −80 °C, respectively.

### 2.2. FISH

For FISH, FFPE sections of infected and control GPSE were baked at 52 °C for 2 h and then deparaffinized and rehydrated according to standard protocols (ending with an *aqua dest.* rinse). Slides were overlaid with 0.2 N hydrochloric acid solution at room temperature (RT) for 20 min and then rinsed again (*aqua dest.*). Thereafter, slides were incubated in 2 × saline sodium citrate (SSC, stock 20 ×: 3 M NaCl, 300 mM trisodium citrate, pH 7) with 0.05% Tween 20 for 5 min at RT and then in 2xSSC for 20 min at 80 °C. Slides were rinsed in *aqua dest.* and again incubated in 2 × SSC with 0.05% Tween 20 (5 min, RT). A digestion step using Proteinase K (20 µg/mL in 20 mM Tris-HCl, pH 7.4, cat. no. 1.24568.0100, Merck, Darmstadt, Germany) for 10 min at 37 °C ensued. Another incubation in 2 × SSC with 0.05% Tween 20 (5 min, RT) was followed by the dehydration of the slides using 70%, 80% and 90% Ethanol successively (2 min each). Thereafter, probes were added (20 ng/µL in 10 mM Tris-HCl and 1 mM EDTA, pH 8; see Table 1) and the specimens were covered with a coverslip. Denaturation of the nucleic acid strands at 90 °C in a humid chamber for 15 min and the over-night hybridization at 42 °C (humid chamber) followed. The next day, coverslips were removed by incubation in 2 × SSC (10 min, 42 °C). Two changes of 2 × SCC with 0.05% Tween 20 for 5 min each at RT ensued. Another washing step (2 × SSC, 5 min, 42 °C) preceded the counterstain using bisBenzimide 33,342 (1:500 in PBS, 20 min, dark; Hoechst, Merck). Finally, the slides were washed multiple times in PBS, covered with coverslips and examined using a Nikon Eclipse Ni microscope equipped with ProgRes CF cool camera and ProgRes Capture Pro 2.8.8 software (all Jenoptik, Jena, Germany).

The specific probes for Gp, *T. benhamiae* and the virulence factors of interest were deduced from nucleotide sequences deposited in the NCBI Blast database. Kingdom-spanning probes, i.e., EUB 388 (detecting eubacteria [28]), EUK 516 (eukaryotes [28]) and PanF (panfungal [17,19]), and specific ones for *Escherichia (E.) coli* [29] and Green Fluorescent Protein (GFP) were used as positive (pos) and negative (neg) controls, respectively. All probes were manufactured by BioTeZ Berlin-Buch GmbH (Berlin, Germany).

Incubation times and temperatures as well as components and concentrations of buffers and probes were modified in different optimization trials (e.g., probe concentrations ranged between 10–40 ng/µL, probes were also diluted in a different buffer consisting of 10% Dextran sulfate w/v, 5 M NaCl, 0.5 M EDTA, 100 mM Tris-HCl, 2 mg/mL bovine serum albumin, 100 μg/mL polyadenosine, 20 μg/mL salmon DNA and 1:500 10% sodium dodecyl sulfate, pH 8; all Sigma (St. Louis, MO, USA) by Merck). Further, different fixatives (e.g., Carnoy, paraformaldehyde (PFA, 4% buffered), Ethanol, Methanol) and a subset of various enzymes for the digestion pretreatment were tested (e.g., lysozyme solution (10 mg/mL in 50 mM Tris-HCl, pH 9, and 20 mM EDTA; Merck), Pepsin Reagent (ready-to-use, cat. no. R2283, Sigma by Merck) and Lysing Enzymes, cat. no. L1412G, Sigma by Merck); detailed information is available upon request.

### 2.3. RNA Isolation and qPCR

For qPCR, one frozen GPSE per condition (time point and fungal isolate) was lyo-philized in a freeze-dryer (Alpha 1-2, Martin Christ^TM^, Osterode, Germany) and stored at 4 °C until further usage.

Explants were removed from lyophilization vials or reaction tubes, respectively, and transferred to sterile single-use petri dishes supplied with peqGOLD TriFast^TM^ (cat. no. 30-2010, Peqlab by vwr) for RNA isolation. GPSE were cut into as of small pieces as possible with single-use scalpels and sterile forceps. The skin pieces were transferred to another conical 1.5 mL reaction tube and 500 µL of TriFast^TM^ were added. Samples were further disrupted using micropestles and homogenized by pipetting (approx. 30 times with a cut 1000 µL tip). Thereafter, 100 µL chloroform were added, the tubes were shaken vigorously for 15 s and incubated at RT for 3–10 min. Final phase separation was achieved by centrifuging (5 min, 12,000× *g*). The RNA containing aqueous phase was transferred to mini spin columns of the RNeasy Mini Kit (cat. no. 74104, Qiagen) and purified according to the manufacturer’s instructions including an on-column DNase I digestion (RNase-free DNase Set, cat. no. 79254, Qiagen). RNA was eluted in 30 µL RNase-free water. Concentration and purity of these RNA preparations were determined spectrophotometrically using an Eon Reader Take3 (absorbance ratio A260/280; Biotek, Winooski, VT, USA); the RNA integrity of selected samples was checked visually using gel electrophoresis. All RNA preparations were stored at −80 °C until further usage. Equally handled, native and time-matched uninfected GPSE served as biological negative controls.

RNA samples were reverse transcribed according to the manufacturer’s instructions of the QuantiTect Reverse Transcription Kit (cat. no. 205311, Qiagen). Volumes of each component of the reaction mixture were adapted to a total volume of 12 µL or 500 ng RNA per sample, respectively (total volume including reverse transcriptase and buffer 20 µL). Reverse transcription controls were included in each run (transcriptase component replaced by H_2_O).

qPCR protocols for four virulence factors, namely *sub 3, sub 6*, *mcpA* and *isocitrate lyase (isol)* were established; the *ADP-ribosylation factor (ADPrf)* served as reference gene for subsequent normalization. The corresponding primer sequences were taken from the literature [5,30]; primer molecules were synthesized by biomers.net (Ulm, Germany).

After thorough optimization and quality control of the PCR protocols, all sample reactions were performed in a total volume of 20 µL using the QuantiNova SYBR Green PCR Master Mix Kit (cat. no. 208054, Qiagen). Each reaction mixture included 10 µL master mix (2×), 1.5 µL forward and reverse primer each (10 µmol), 2 µL H_2_O and 5 µL freshly diluted cDNA (1:10 in DNAse-free H_2_O). All virulence factor reactions were carried out identically on the RotorGeneQ system (Qiagen) with the following thermal profile: after 2 min at 50 °C, the initial denaturation for 10 min at 95 °C followed. After that, 40 cycles of denaturation for 15 s at 95 °C and combined annealing and elongation for 30 s at 60 °C ensued (for *ADPrf*: 10 s at 95 °C and 15 s at 58 °C for 40 cycles). The obtained fluorescence signals were measured after each cycle in the green channel. A melting curve of the qPCR products from 70 to 90 °C with 1°C increments concluded each run. Reverse-transcription- and non-template-controls (NTCs) as technical negative controls (Cq values > 40 were considered negative) were always included. 

All samples were run in single per primer pair alongside a six-point standard curve, i.e., a dilution series of pJET1.2/blunt-plasmids containing the respective amplicon of each gene of interest (10^7^ to 10^−1^ molecules/µL). The plasmids were assembled using the CloneJET PCR Cloning Kit (cat. no. K1232, Thermo Scientific, Dreieich, Germany) including a blunting reaction and were transformed into *E. coli* XL 10 Gold via electroporation for amplification. The correct insertion of the amplicons was verified by sequencing using the primers provided by the manufacturer; plasmids were isolated from liquid *E. coli* cultures using the QIAprep Spin Miniprep Kit (cat. no. 27104, Qiagen) and stored at 4 °C. They also served as technical positive controls and enabled further quality control of each run, i.e., reactions were considered valid if their efficiency was within a 5% deviation compared to the previously determined value. Efficiency as well as other information about the qPCRs established during this study are given in Table 2.

Cq values of samples and controls were determined by the RotorGeneQ Software 2.0.0 (Qiagen) at a threshold of 0.1 normalized fluorescence units and automatically converted into absolute transcript numbers. Final transcript numbers are given normalized to 10^3^ *ADPrf* molecules/reaction which reduces the impact of variations during RNA isolation, reverse transcription and qPCR amplification (“calibrated quantification” [23]).

### 2.4. Statistics

The obtained data were statistically analyzed using SigmaStat 2.03 and SigmaPlot 7.0 software (Systat Software, Erkrath, Germany). Student’s t test and Mann–Whitney U test were employed to compare infected and native GPSE, *T. benhamiae* isolates derived from humans and Gp and transcript levels and time points, respectively. *p* values of less than 0.05 were considered significant and results were presented as mean ± standard deviation or median, respectively.

## 3. Results

### 3.1. FISH

**Figure 1:**
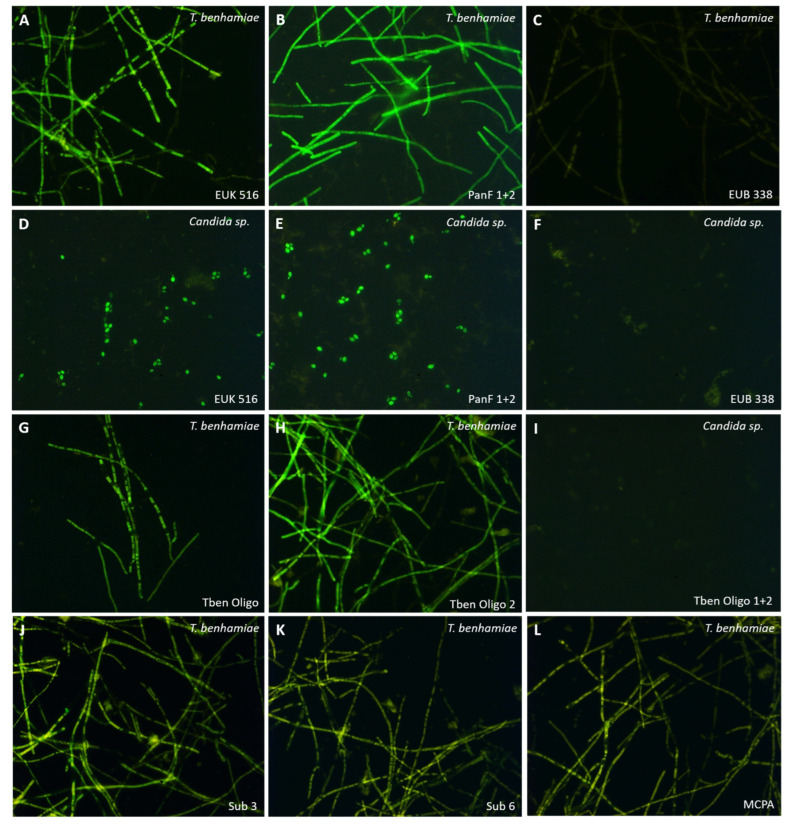
FISH in *T. benhamiae* isolates and controls (*Candida* sp.). The fungal species is indicated in the upper right corner and the used probe/probe mix (all coupled to 6-FAM and shown in green) in the lower right corner of each picture. First and second row (**A**–**F**): the eukaryote (EUK 516) and the panfungal probes (PanF 1 + 2) stain positive while the eubacteria probe (EUB 388) stains negative in both fungal species used. Third row (**G**–**I**): Oligo 1 (Tben) and 2 (Tben) were specifically designed for *T. benhamiae;* probe specificity is proven by negative staining of *Candida* sp. The fourth row (**J**–**L**) shows virulence factor-specific probes staining positive in different *T. benhamiae* isolates (all images: original magnification 400×).

The specificity of control and species-specific probes was demonstrated in several in vitro trials with *T. benhamiae* hyphae and *Candida* sp. yeast cells fixed to object slides (Figure 1), Gp fibroblasts derived from freshly excised GPSE and native Gp skin sections respectively. None of the used fixatives proved superior leading to the decision to stick to the well-established 24 h PFA fixation. A prolonged enzymatic pretreatment using any one of the tested enzymes (e.g., Proteinase K, Pepsin Reagent, lysozyme solution etc.) proved essential to break open fungal cell walls whereas for fibroblasts 10 min of permeabilization using the nonionic detergent Triton® X-100 dissolved in PBS was sufficient for successful hybridization. Short term or over-night hybridization as well as simple vs. more-component probe diluents did not make a difference in terms of signal strength.

As expected, the probes detecting eukaryotes (i.e., EUK 516 and PanF 1 + 2) were labeled *T. benhamiae* and *Candida* sp. equally well (Figure 1A,B,D,E), while EUB 388 served as negative control for fungi (Figure 1C,F; positive staining of *E. coli* not shown). Successful hybridization resulted in fluorescence of the fungal cytoplasm in coccoid yeast cells as well as in filamentous hyphal structures. However, staining signals were quite homogenous in the former but more variable along *T. benhamiae* hyphae. This staining pattern was also seen with the species-specific probes for *T. benhamiae* (Oligo 1 (Tben) and 2 (Tben); Figure 1G,H) and the virulence factor-specific probes directed against *sub 3*, *sub 6* and *mcpA* (Figure 1J–L); Oligo 1 and 2 (Tben) did not hybridize with *Candida* sp. cells (Figure 1I).

**Figure 2:**
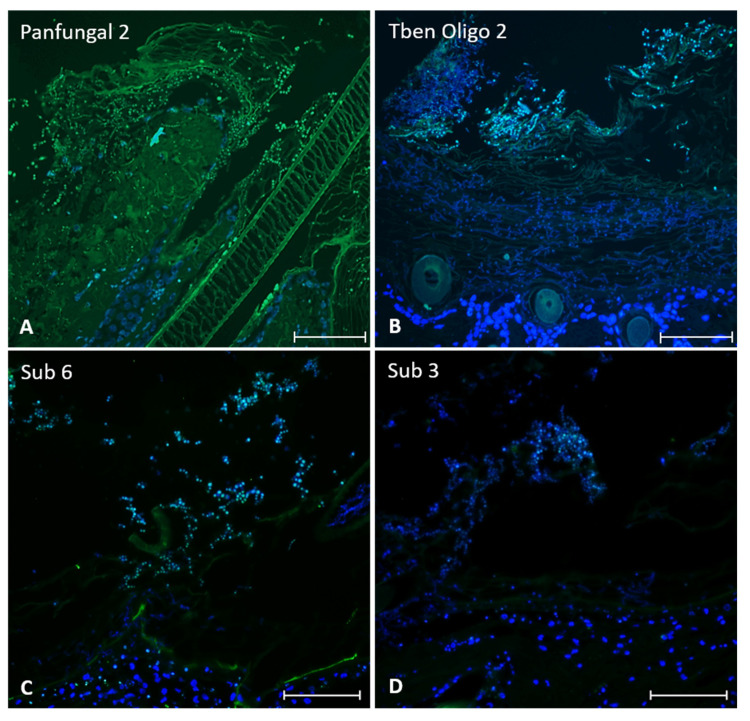
FISH in *T. benhamiae* infected GPSE. (**A**) *T. benhamiae* infected GPSE were incubated with the “Panfungal 2” probe (shown in green); fungal elements in the *stratum corneum*, mainly conidia, are stained. In (**B**), the *T. benhamiae* specific probe “Oligo 2 (Tben)” clearly marks the conidia while hyphae infiltrating the *stratum corneum* and deeper skin layers show a less intense staining. In (**C**,**D**), the probes detecting the virulence factor transcripts *sub 6* and *3*, respectively, mainly label conidia in superficial epidermal layers (all images: original magnifications 200×, bars = 100 µm; nuclear counterstain ensued with bisBenzimide Hoechst 33342 and is depicted in blue).

For FFPE sections of *T. benhamiae* infected and control GPSE, only inconsistent stainings were achieved. During protocol optimization, at times quite convincing staining patterns were observed as exemplified in Figure 2, but those results could not be reproduced reliably. In some cases, a high background staining, i.e., unspecific staining and autofluorescence exhibited by Gp *stratum corneum* and hair (Figure 2A), were observed.

### 3.2. RNA Isolation and qPCR

Several methods for tissue disruption and RNA release were tested including ultra-sonication, deep freezing, grinding in liquid nitrogen and bead beating in a tissue homogenizer. However, concentration (0–153.95 ng/µL) and purity (A260/280: 1.4–2.6) of RNA preparations varied considerably regardless of the employed method. It is noteworthy that using RNAprotect Tissue Reagent (Qiagen, Hilden, Germany) did not improve RNA yield either. Standardizing the employed RNA template in terms of concentration, using optimal conditions for reverse transcription and qPCR as well as the choice of a suitable reference gene were measures to keep the impact of this variability on the overall result as small as possible.

A total of 164 qPCR results (41 infected GPSE, at least six biological replicates per sampling day and virulence factor) were considered in the following analysis. Generally, the expression of the genes of interest was demonstrated for all employed *T. benhamiae* isolates. As expected, the biological negative controls, i.e., native GPSE, were negative for all investigated genes since they are not found in mammalian species [31]. NTCs were negative throughout all considered reactions and melting curve analysis revealed no unspecific qPCR products (data not shown).

*sub 3*-transcripts were found in a range of 2.73 × 10^1^–2.51 × 10^5^/10^3^ *ADPrf*-transcripts with the highest expression by a Gp derived isolate on d 10 (isolate TbMS8A). In addition, for Gp derived isolates, a significant increase of the *sub 3*-expression rate toward the end of culture was found (d 3 vs. d 10: *p* = 0.017, d 5 vs. d 10: *p* = 0.001). For *mcpA*-transcripts, a range of 1.1 × 10^2^–2.2 × 10^4^/10^3^ *ADPrf*-transcripts was determined. The highest expression was seen in a human derived isolate on d 7 (isolate Tbhum207860). *sub 6*-transcript numbers ranged between 1.48 × 10^1^–5.14 × 10^4^/10^3^ *ADPrf*-molecules. The highest expression was exhibited by a human derived isolate on d 3 (isolate Tbhum206494). *Isol*-transcripts ranged from 2.57 × 10^1^–7.14 × 10^3^/10^3^ *ADPrf*-molecules (highest: d 7, TbMS5A). Both isolate groups expressed *isol* throughout the culture at a comparatively low level, with a significant decrease toward d 10 by Gp derived isolates (d 3 vs. d 10: *p* = 0.017). Comparing the sampling time points, for human derived isolates no change of the expression rates of all virulence factors was seen. In addition, there was no difference between the two groups of isolates at any time point for all four virulence factors. 

Human derived *T. benhamiae* isolates produced significantly more *sub 3* than *sub 6* on d 7 (*p* = 0.004) and d 10 (*p* = 0.029) of culture. On d 10, more *mcpA* was found than *sub 6* (*p* = 0.029). *isol*-transcripts were found significantly less on d 10 compared to the other virulence factors (*mcpA*: *p* = 0.029; *sub 6*: *p* = 0.019; *sub 3* d 7: *p* = 0.015, d 10 *p* = 0.029; see Supplementary Table S3). In GDP derived isolates, significantly more *sub 3* than *sub 6*, *mcpA* and *isol* was found on d 7 and d 10. In addition, on these sampling time points, significantly less *sub 6*-transcripts were found compared to *mcpA* (for *p* values, see Supplementary Table S4).

Considering all isolates regardless of origin as one group (*n* = 6–13), the abovementioned findings are even extrapolated: there is a significant increase of *sub 3*- and *mcpA*-transcripts toward the end of culture (*sub 3*: d 3 vs. d 10: *p* = 0.001, d 5 vs. d 10: *p* < 0.001; *mcpA*: d 3 vs. d 10: *p* = 0.014, d 5 vs. d 10: *p* = 0.001, d 7 vs. d 10: *p* = 0.032). The number of *isol*-transcripts decreased significantly (d 3 vs. d 10: *p* = 0.014). *sub 3* was expressed significantly more than all other virulence factors from d 5 onwards. Less *sub 6* was found compared to *mcpA* on d 7 and d 10 (for *p* values, see Supplementary Table S5).

**Figure 3:**
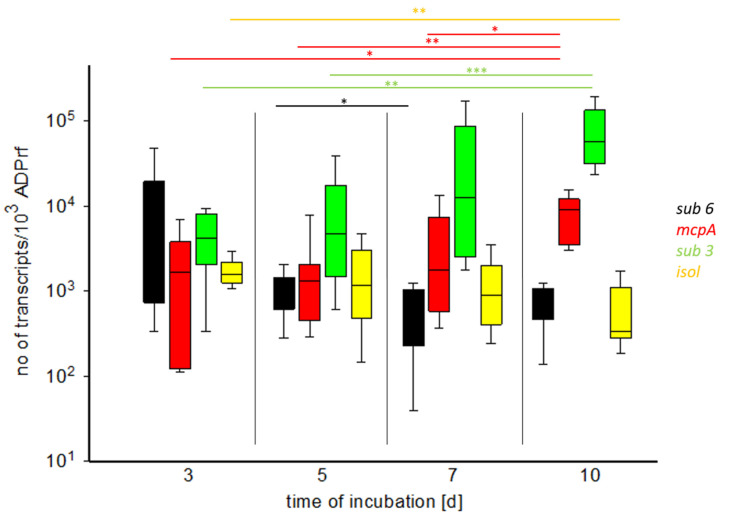
Expression rates of the virulence factor transcripts *sub 3* (green), *sub 6* (black), *mcpA* (red) and *isol* (yellow) of *T. benhamiae* isolates in GPSE culture from d 3 to d 10 (normalized to 10^3^ *ADPrf*-transcripts). A significant increase of *sub 3*- and *mcpA*-transcripts toward the end of culture was noticed. On the contrary, the number of *isol*-transcripts decreased significantly. Furthermore, *sub 3* and *mcpA* were significantly more abundant than *sub 6* and *isol* (Man-Whitney-U test, * *p* < 0.05, ** *p* = 0.001, *** *p* > 0.001).

The median expression rates of all examined virulence factors per sampling day are given in Table 3 and visualized in Figure 3 (expressed as number of mRNA-transcripts per 10^3^ *ADPrf*-transcripts; “calibrated qPCR” [23]). 

## 4. Discussion

### 4.1. FISH

Although widely applied in a variety of research areas and specimens [32], there are no reports of dermatophytes being detected in skin samples using FISH. Only one study describes an in situ hybridization technique using genomic probes (GISH) to identify the causative agents of clinically confirmed dermatophytoses, namely *T. interdigitale, T. rubrum* and *M. canis* [33]. Worek et al. isolated and cultured clinical dermatophyte samples and subjected isolated hyphae to GISH, which was successfully carried out in our lab as well (see Figure 1). Unfortunately, the exact target of the probe mix used in their study is not known since the probes were based on DNA extracts from reference strains without further sequence analysis. The authors found false-positive hybridization signals in less than 10% of the tested samples but stated that such signals cannot be ruled out, especially with the closely related *Trichophyton* spp. Consequently, the authors conclude that the probes are “fairly specific” and recommend this method rather as a supplement to the PCR-based dermatophyte identification in ambiguous cases [33].

Here, we report inconclusive stainings and missing reproducibility of FISH in GPSE. We thoroughly tested our probes by showing their functionality in isolated hyphae and modified our protocols in numerous ways but results remained inconsistent. Biological reasons most likely include, but are not limited to, the number, distribution and conformation of the target sequence, differences in the cell wall permeability along hyphae, autofluorescence of other structures and necrotic areas in the specimen [29,34]. As for the number of target sequences, most studies pursued species identification using rRNA targets which are abundantly found in ribosomes [35]. Specific mRNA transcripts are less frequent and tightly regulated surely influencing a FISH-based detection [36,37]. There is also a considerable number of technical setscrews of FISH, e.g., specimen pretreatment, hybridization temperatures, post-hybridization washing conditions, probe characteristics etc. (for details see [32] and ref. therein). Since the probes used herein hybridized successfully with isolated fungal elements, we do not doubt their suitability. On the contrary, fungal cell walls display compact structures which are difficult to digest enzymatically or to break down chemically [38,39]. Moreover, in the ex vivo model, fungal elements were embedded in skin tissue which exacerbates this situation.

A worthwhile future approach might also be the usage of probe mixes spanning the entire length of the target sequence in terms of signal enhancement. The latter might also be achieved using a catalyzed reporter deposition methodology (CARD) due to a higher sensitivity and a superior spatial resolution of the staining which is especially important when only few copies of the target sequence are expected [40,41].

### 4.2. qPCR of Virulence Factors

As outlined before, fungal cell walls and skin are very compact structures that are not easily prepared for microbiological applications. Therefore, a combination of different tissue disruption methods was expected to work best for sufficient dermatophyte RNA isolation from GPSE. Taken together, the usage of lyophilized, cut and ground GPSE, TriFast^TM^ and a column-based RNA purification method was found acceptable.

To the best of our knowledge, this is the first study absolutely quantifying transcript numbers of the important dermatophyte virulence factors *sub 3*, *sub 6, mcpA* and *isol*. The lack of quantitative data and detailed kinetics on these virulence factors, especially in vivo, hampers the assessment of our results for the time being [4,11]. Furthermore, the still changing *Trichophyton* nomenclature needs to be followed closely. For example, the NCBI BLASTn acc. no. “LN874022.1” used to belong to *T. benhamiae* “white strains”. Recently, Čmoková et al. suggested the corresponding isolate (DMF2446) to be categorized as the newly described species *T. japonicum* based on an elaborate polyphasic approach to resolve the taxonomy of the closely related members of the *Trichophyton* clade [42]. This multifaceted strategy will probably have the power to reorganize dermatophyte taxonomy in general.

In our study using ex vivo GPSE, *sub 3*-transcripts were most abundant, although Sub 3 protease was previously found predominantly in vitro. For example, *M. canis*-Sub 3 was shown to be the major proteinaceous component secreted into a minimal liquid medium containing cat hair as the sole nitrogen source [43]. *T. rubrum* conidia showed a 10-fold increase of *sub 3*-transcripts after 24h when grown on keratin substrates [44]. Likewise, Staib et al. found *sub 3*-transcripts upregulated during growth of *T. benhamiae* in Sabouraud medium and only “at a comparatively low level” in experimental Gp infection [5]. We ascribe the abundance of *sub 3* in our GPSE experiments to the de novo formation of conidia which were numerously formed during tissue culture around d 7. First, this assumption is underlined by our previous IF study at protein level confirming a strong Sub 3 expression in conidia (“capping effects” [10]). Second, *sub 3*-transcripts peaked from d 7 onwards coinciding with the described release of fully matured conidia [45]. Furthermore, Sub 3 proteases in conidia were also shown in skin sections of cattle naturally affected by trichophytosis (causative agent: *T. verrucosum* [46]). Noteworthy, the latter identified Sub 3 exclusively in conidia; others found it also in hyphae [43], as shown in our study. Belgian investigators detected the protease in *M. canis* conidia using RNA silencing experiments thereby proving an essential role of Sub 3 for conidial adhesion to epidermal structures of different hosts [13,14]. The importance of serine and subtilisin-like proteases for adhesion to and initial colonization of different host tissues was also shown in other fungal, bacterial and protozoan pathogens [14] (and refs. therein). Moreover, the high keratinolytic activity of Sub 3 seems to be a favorable attribute for conidia in terms of infection initialization given the fact that dermatophytes almost exclusively develop in keratinized tissues [47]. 

As with the subtilisins, there is only limited in vivo data available on *mcpA*/MCPA. Nevertheless, Zaugg and colleagues revealed an intriguing connection between subtilisin-like proteases and MCPA [48]: they demonstrated the activation of *T. rubrum* MCPA through the proteolytic removal of its propeptide almost exclusively by the proteases Sub 3 and/or Sub 4 being simultaneously released into a proteinaceous medium. In that sense, the concomitant expression and increase of *sub 3-* and *mcpA*-transcripts toward the end of the current ex vivo tissue culture seems reasonable. 

The functional analysis of MCPA using closely related microorganisms or such with a similar mode of life is challenging since to date no orthologous peptidases in yeasts or related filamentous ascomycetes have been identified [48]. In *Aspergillus* spp., only one gene sequence probably encoding a related secreted metalloprotease of the M14 protein family was found, namely MCPB, but it is still considered putative and remains an unassigned protease. In *T. benhamiae*, several putative *mcpA* gene copies were deduced, namely ARB_07026/ARB_07027 [49] and ARB_03789 (Burmester et al., unpublished data). However, whether all putative gene copies are transcribed into (functional) proteins is also not known (note: the herein used primer set (mcpA-f/mcpA-rev [30]) recognizes ARB_07026, a hypothetical protein of *T. verrucosum* with M14 protein family characteristics (XM_003017997.1), *T. rubrum* EU024297.1 and XM_003235075.1 (TERG_04176) (both “secreted carboxypeptidase McpA gene”) according to NCBI BLastn). The fact that *mcpA*/MCPA was demonstrated in vitro [6,30], in vivo [5] and with the herein presented data also ex vivo might support the postulated importance and versatility of this enzyme [30].

The serine protease Sub 6 is considered a putative in vivo disease marker of dermatophyte infections since it was identified in onychomycosis patients affected by *T. rubrum* and after experimental infection of Gp with *T. benhamiae* [5,6,15,50]. Moreover, Gp infected with a *sub 6* deletion mutant of *T. mentagrophytes* showed a delayed onset of clinical symptoms, less severe pathological changes and lower immunological reactions compared to the control group [51]. The Sub 6 protease was also found to be immunogenic and to induce both IgE-antibody and cell-mediated immune responses in humans [52]. However, expression kinetics and absolute secretion levels during host infection are not known to date. 

Only one study investigated kinetics of the expression of *T. rubrum* secreted proteases in the presence of full-thickness human skin explants over a time span of 24h [12]. All five fungalysins [53] and all seven subtilisins [47] described in *Trichophyton* spp. were demonstrated in this set up by the authors; increasing transcript numbers were seen for *sub 3, sub 4, mep 3* and *mep 4* toward the end of the observed culture time. *sub 6* was found at a low level throughout the skin explant co-culture, as in the current study, and, likewise, when explants were replaced by keratin, elastin or collagen powder representing the major constituents of epidermis and dermis, respectively. This low expression is explained by Leng and colleagues with the presence of other factors that might suppress protease expression or dermatophyte growth altogether (e.g., 2-macroglobulin keratinase inhibitor, unsaturated transferrin etc.) [12]. Taken together, these results suggest *sub 6* to play a rather subordinate role in the described ex vivo systems.

Isocitrate lyase is a key enzyme of the anaplerotic glyoxylate cycle that catalyzes the cleavage of isocitrate to succinate and glyoxylate and, thus, enables the net assimilation of carbon from two-carbon compounds rather than simple carbohydrates. Dermatophytes colonize highly specific host niches where proteinaceous substrates predominate; therefore, such alternative metabolic pathways are required to ensure gluconeogenesis and other biosynthetic pathways [31]. Hence, key enzymes of this pathway are discussed to contribute to virulence and/or host adaptation [5]. In that sense, its expression throughout GPSE culture seems reasonable. Staib et al. found *isol* slightly upregulated during experimental Gp infection compared to growth on Sabouraud agar but only two animals were sampled at different time points [5]. In *T. rubrum*, but not in *T. benhamiae*, grown in soy and keratin–soy media *isol* was strongly upregulated [30]. These data rather support the idea of a naturally occurring differential expression of the proteases among the different dermatophyte species [48,54,55,56]. Altogether, this strongly implies further comparative research before conclusively determining the role of certain virulence attributes in a distinct context.

## 5. Conclusions

As indicated before, few studies have investigated the expression and kinetics of important dermatophyte virulence factors to date. However, a direct comparison is not feasible due to several varying parameters such as deviating time spans, gene vs. protein level investigations, different experimental systems, dermatophyte species and controls, etc. In that sense, this work adds an important puzzle piece to the knowledge about *sub 3, sub 6*, *mcpA* and *isol* obtained during long-term culture using a versatile ex vivo infection model. We strongly encourage further research of this kind—especially when it comes to quantitative data—to finally elucidate the specific role of each of the virulence factors during real in vivo infection and disease.

## Figures and Tables

**Table 1:** Name, sequence and further information about the probes used for FISH.

Probe	Target Gene (NCBI acc. no.)	Dye	Sequence (5′-3′)	Tm [°C]	Ref.
Oligo1 (Tben)	18S rRNA *T. benhamiae* (AY083225.1)	6FAM	CCATGTAGTAAGGTACTATCAA	60	own
Oligo2 (Tben)	18S rRNA *T. benhamiae* (AY083225.1)	6FAM	TTCGGCAAATCCAAGAATTTCA	60	own
Oligo3 (Gp)	18S rRNA Guinea pig (AAKN02059112.1)	Cy3	TACTACCGATTGGATGGTTTAG	62	own
Oligo4 (Gp)	18S rRNA Guinea pig (AAKN02059112.1)	Cy3	TCTTAGTTGGTGGAGCGATTTG	64	own
sub3-f	*sub3* (AY437854.1)	6FAM	GAGCAACGCTAACACCCTGGGCAAGCATG	82	own
sub3-taq	*sub3* (AY437854.1)	6FAM	CAATCTGCTTCAAGCGGTCGCAGGCCT	86	own
sub6-f	*sub6* (AY437857.1)	6FAM	TACCAGAGAGAGTATCAGTGCTGCCGC	84	own
sub6-taq	*sub6* (AY437857.1)	6FAM	CCGCAAACGTGAGGAGAAGCCATGGAAG	88	own
mcpA-f	*mcpA* (XM_003014418.1)	6FAM	GGAGTTCCATGCACCGCCTTCAATGC	82	own
mcpA-taq	*mcpA* (XM_003014418.1)	6FAM	GGTAGATGGTGTTGCAGATGGGCCCGG	88	own
GFP	Green Fluorescent Protein (neg) (MN513050.1)	6FAM	GAGTTAAAAGGTATTGATTTTAAAG	88	own
Prev	16S rRNA *Escherichia coli* (neg)	FITC	CCACATGTTCCTCCGCTTGT	62	[29]
PanF-1	fungi (pos)	6FAM	CCGATCCCTAGTCGGCATAG	62	[19]
PanF-2	fungi (pos)	6FAM	CTCTGGCTTCACCCTATTC	58	[17]
EUB 338	16S rRNA eubacteria (neg)	6FAM	GCTGCCTCCCGTAGGAGT	60	[28]
EUK 516	18S rRNA eukaryotes (pos)	6FAM	ACCAGACTTGCCCTCC	52	[28]

Tm—melting temperature, 6FAM—6-carboxyfluorescein, Cy3—cyanine 3, FITC—fluorescein isothiocyanate, pos—positive control, neg—negative control.

**Table 2:** Characteristics of the established qPCRs.

Gene of Interest [5]	Plasmid (pJET1.2/Blunt; 2974 bp)	Linearity [Molecules/Reaction]	Sensitivity [Molecules/ Reaction]	Efficiency (Validity ± 5%)	Tm Product [°C]
*Isocitrate lyase* *(isol)*	pJET1.2/blunt-Isol (3077 bp)	50	5	1.03	80.2
*Metallocarboxy-peptidase A (mcpA)*	pJET1.2/blunt-MCPA (3058 bp)	500	5	0.94	80.7
*Subtilisin 3 (sub 3)*	pJET1.2/blunt-Sub3 (3055 bp)	50	5	0.99	84.0
*Subtilisin 6 (sub 6)*	pJET1.2/blunt-Sub6 (3095 bp)	50	5	1.00	82.3
Reference gene: *ADP—ribosylation factor (ADPrf)*	pJET1.2/blunt-ADPRF (3048 bp)	50	5	0.91	80.2

**Table 3:** Median expression rates of the virulence factors *sub 3*, *sub 6*, *mcpA* and *isol* of *T. benhamiae* isolates in GPSE culture from d 3 to d 10 (normalized to 10^3^ *ADPrf*-transcripts).

Sample Day	*T. benhamiae* Isolates			
	*sub 3*	*sub 6*	*mcpA*	*isol*
3 (*n* = 6)	4.19 × 10^3^	1.63 × 10^3^	1.65 × 10^3^	1.57 × 10^3^
5 (*n* = 11)	4.76 × 10^3^	1.14 × 10^3^	1.33 × 10^3^	1.16 × 10^3^
7 (*n* = 13)	1.25 × 10^4^	6.04 × 10^2^	1.75 × 10^3^	9.03 × 10^2^
10 (*n* = 11)	5.75 × 10^4^	8.99 × 10^2^	9.17 × 10^3^	3.36 × 10^2^

## Supplementary Materials

The following are available online at https://www.mdpi.com/article/10.3390/jof8010024/s1, Table S1, Further information on *T. benhamiae* isolates used during study. Table S2, Name, sequence and further information on the primers used for PCR and qPCR during this study. Table S3, The expression rates of the different virulence factors found in human derived *T. benhamiae* isolates were compared (*p* values; statistically significant differences are defined as *p* < 0.05 and indicated in bold letters). Table S4, The transcript numbers of the different virulence factors expressed by Gp derived *T. benhamiae* isolates were compared (*p* values; statistically significant differences are defined as *p* < 0.05 and indicated in bold letters). Table S5, The expression rates of the different virulence factors produced by *T. benhamiae* isolates were compared (*p* values; statistically significant differences are defined as *p* < 0.05 and indicated in bold letters).

## References

[1] Havlickova B., Czaika V.A., Friedrich M. (2008). Epidemiological trends in skin mycoses worldwide. Mycoses.

[2] Saunte D.M.L., Hare R.K., Jørgensen K.M., Jørgensen R., Deleuran M., Zachariae C.O., Thomsen S.F., Bjørnskov-Halkier L., Kofoed K., Arendrup M.C. (2019). Emerging Terbinafine Resistance in *Trichophyton*: Clinical Characteristics, Squalene Epoxidase Gene Mutations, and a Reliable EUCAST Method for Detection. Antimicrob. Agents Chemother..

[3] Ebert A., Monod M., Salamin K., Burmester A., Uhrlaß S., Wiegand C., Hipler U.-C., Krüger C., Koch D., Wittig F. (2020). Alarming India-wide phenomenon of antifungal resistance in dermatophytes: A multicentre study. Mycoses.

[4] Achterman R.R., White T.C. (2012). Dermatophyte Virulence Factors: Identifying and Analyzing Genes That May Contribute to Chronic or Acute Skin Infections. Int. J. Microbiol..

[5] Staib P., Zaugg C., Mignon B., Weber J., Grumbt M., Pradervand S., Harshman K., Monod M. (2010). Differential gene expression in the pathogenic dermatophyte *Arthroderma benhamiae* in vitro versus during infection. Microbiology.

[6] Tran V.D.T., De Coi N., Feuermann M., Schmid-Siegert E., Băguţ E.-T., Mignon B., Waridel P., Peter C., Pradervand S., Pagni M. (2016). RNA Sequencing-Based Genome Reannotation of the Dermatophyte *Arthroderma benhamiae* and Characterization of Its Secretome and Whole Gene Expression Profile during Infection. mSystems.

[7] Russell W.M.S., Burch R.L. (1959). The Principles of Humane Experimental Technique.

[8] Quatrin M.P., Flores Dalla Lana D., Andrzejewski Kaminski T.F., Meneghello Fuentefria A. (2019). Fungal infection models: Current progress of ex vivo methods. Mycoses.

[9] Baumbach C., Schrödl W., Nenoff P., Uhrlaß S., Mülling C.K.W., Michler J.K. (2020). Modeling dermatophytosis: Guinea pig skin explants represent a highly suitable model to study *Trichophyton benhamiae* infections. J. Dermatol..

[10] Baumbach C., Michler J.K., Nenoff P., Uhrlaß S., Schrödl W. (2020). Visualising virulence factors: *Trichophyton benhamiaes* subtilisins demonstrated in a guinea pig skin ex vivo model. Mycoses.

[11] Monod M. (2008). Secreted Proteases from Dermatophytes. Mycopathologia.

[12] Leng W., Liu T., Wang J., Li R., Jin Q. (2009). Expression dynamics of secreted protease genes in *Trichophyton rubrum* induced by key host’s proteinaceous components. Med. Mycol..

[13] Baldo A., Mathy A., Tabart J., Camponova P., Vermout S., Massart L., Maréchal F., Galleni M., Mignon B. (2010). Secreted subtilisin Sub3 from *Microsporum canis* is required for adherence to but not for invasion of the epidermis. Br. J. Dermatol..

[14] Băguţ E.T., Baldo A., Mathy A., Cambier L., Antoine N., Cozma V., Mignon B. (2012). Subtilisin Sub3 is involved in adherence of Microsporum canis to human and animal epidermis. Vet. Microbiol..

[15] Méhul B., De Coi N., Grundt P., Genette A., Voegel J.J., Monod M. (2019). Detection of *Trichophyton rubrum* and *Trichophyton interdigitale* in onychomycosis using monoclonal antibodies against Sub6 (Tri r 2). Mycoses.

[16] Amann R., Fuchs B.M. (2008). Single-cell identification in microbial communities by improved fluorescence in situ hybridization techniques. Nat. Rev. Genet..

[17] Kempf V.A.J., Trebesius K., Autenrieth I.B. (2000). Fluorescent In Situ Hybridization Allows Rapid Identification of Microorganisms in Blood Cultures. J. Clin. Microbiol..

[18] Martins M.L., Ferreira A., Sampaio A., Vieira R., Inácio J. (2010). Direct and specific identification of *Cryptococcus neoformans* in biological samples using fluorescently labelled DNA probes. Eur. J. Clin. Microbiol. Infect. Dis..

[19] Hayden R.T., Isotalo P.A., Parrett T., Wolk D.M., Qian X., Roberts G.D., Lloyd R.V. (2003). In Situ Hybridization for the Differentiation of *Aspergillus*, *Fusarium*, and *Pseudallescheria* Species in Tissue Section. Diagn. Mol. Pathol..

[20] Montone K.T., Livolsi V.A., Lanza D.C., Kennedy D.W., Palmer J., Chiu A., Feldman M.D., Loevner L.A., Nachamkin D.I. (2011). In Situ Hybridization for Specific Fungal Organisms in Acute Invasive Fungal Rhinosinusitis. Am. J. Clin. Pathol..

[21] Rickerts V., Smith I.M., Mousset S., Kommedal O., Fredricks D.N. (2013). Deciphering the aetiology of a mixed fungal infection by broad-range PCR with sequencing and fluorescence in situ hybridisation. Mycoses.

[22] Bustin S.A., Benes V., Garson J.A., Hellemans J., Huggett J., Kubista M., Mueller R., Nolan T., Pfaffl M.W., Shipley G.L. (2009). The MIQE Guidelines: Minimum Information for Publication of Quantitative Real-Time PCR Experiments. Clin. Chem..

[23] Svec D., Tichopad A., Novosadova V., Pfaffl M.W., Kubista M. (2015). How good is a PCR efficiency estimate: Recommendations for precise and robust qPCR efficiency assessments. Biomol. Detect. Quantif..

[24] Descamps F., Brouta F., Baar D., Losson B., Mignon B., Monod M., Zaugg C. (2002). Isolation of a *Microsporum canis* Gene Family Encoding Three Subtilisin-Like Proteases Expressed in vivo. J. Investig. Dermatol..

[25] Brouta F., Descamps F., Monod M., Vermout S., Losson B., Mignon B. (2002). Secreted Metalloprotease Gene Family of *Microsporum canis*. Infect. Immun..

[26] Sharma R., Rajak R.C., Pandey A.K., Gräser Y. (2006). Internal Transcribed Spacer (ITS) of rDNA of appendaged and non-appendaged strains of *Microsporum gypseum* reveals *Microsporum appendiculatum* as its synonym. Antonie Leeuwenhoek.

[27] Symoens F., Jousson O., Packeu A., Fratti M., Staib P., Mignon B., Monod M. (2013). The dermatophyte species *Arthroderma benhamiae*: Intraspecies variability and mating behaviour. J. Med. Microbiol..

[28] Amann R.I., Binder B.J., Olson R.J., Chisholm S.W., Devereux R., Stahl D.A. (1990). Combination of 16S rRNA-targeted oligonucleotide probes with flow cytometry for analyzing mixed microbial populations. Appl. Environ. Microbiol..

[29] Rickerts V., Khot P.D., Myerson D., Ko D.L., Lambrecht E., Fredricks D.N. (2011). Comparison of quantitative real time PCR with Sequencing and ribosomal RNA-FISH for the identification of fungi in Formalin fixed, paraffin-embedded tissue specimens. BMC Infect. Dis..

[30] Zaugg C., Monod M., Weber J., Harshman K., Pradervand S., Thomas J., Bueno M., Giddey K., Staib P. (2009). Gene Expression Profiling in the Human Pathogenic Dermatophyte *Trichophyton rubrum* during Growth on Proteins. Eukaryot. Cell.

[31] Dunn M., Ramírez-Trujillo J.A., Hernández-Lucas I. (2009). Major roles of isocitrate lyase and malate synthase in bacterial and fungal pathogenesis. Microbiology.

[32] Young A.P., Jackson D.J., Wyeth R.C. (2020). A technical review and guide to RNA fluorescence in situ hybridization. PeerJ.

[33] Worek M., Kwiatkowska A., Ciesielska A., Jaworski A., Kaplan J., Miedziak B., Deręgowska A., Lewinska A., Wnuk M. (2014). Identification of dermatophyte species using genomic in situ hybridization (GISH). J. Microbiol. Methods.

[34] Rickerts V. (2016). Identification of fungal pathogens in Formalin-fixed, Paraffin-embedded tissue samples by molecular methods. Fungal Biol..

[35] Montone K.T. (2009). Differentiation of *Fusarium* From *Aspergillus* Species by Colorimetric In Situ Hybridization in Formalin-Fixed, Paraffin-Embedded Tissue Sections Using Dual Fluorogenic-Labeled LNA Probes. Am. J. Clin. Pathol..

[36] Houseley J., Tollervey D. (2009). The Many Pathways of RNA Degradation. Cell.

[37] Doma M.K., Parker R. (2007). RNA Quality Control in Eukaryotes. Cell.

[38] Scharf S., Bartels A., Kondakci M., Pfeffer K., Henrich B., Haas R. (2020). Introduction of a bead beating step improves fungal DNA extraction from selected patient specimens. Int. J. Med. Microbiol..

[39] Rodrigues P., Venâncio A., Lima N. (2018). Toxic reagents and expensive equipment: Are they really necessary for the extraction of good quality fungal DNA?. Lett. Appl. Microbiol..

[40] Pernthaler A., Amann R. (2004). Simultaneous Fluorescence In Situ Hybridization of mRNA and rRNA in Environmental Bacteria. Appl. Environ. Microbiol..

[41] Speel E.J., Hopman A.H., Komminoth P. (1999). Amplification Methods to Increase the Sensitivity of In Situ Hybridization: Play CARD(S). J. Histochem. Cytochem..

[42] Čmoková A., Kolařík M., Dobiáš R., Hoyer L.L., Janouškovcová H., Kano R., Kuklová I., Lysková P., Machová L., Maier T. (2020). Resolving the taxonomy of emerging zoonotic pathogens in the Trichophyton benhamiae complex. Fungal Divers..

[43] Mignon B., Swinnen M., Bouchara J.P., Hofinger M., Nikkels A., Pierard G., Gerday C., Losson B. (1998). Purification and characterization of a 315 kDa keratinolytic subtilisin-like serine protease from *Microsporum canis* and evidence of its secretion in naturally infected cats. Med. Mycol..

[44] Bitencourt T.A., Macedo C., Franco M.E., Assis A.F., Komoto T.T., Stehling E.G., Beleboni R.O., Malavazi I., Marins M., Fachin A.L. (2016). Transcription profile of *Trichophyton rubrum* conidia grown on keratin reveals the induction of an adhesin-like protein gene with a tandem repeat pattern. BMC Genom..

[45] Bibel D.J., A Crumrine D., Yee K., King R.D. (1977). Development of arthrospores of *Trichophyton mentagrophytes*. Infect. Immun..

[46] Lindenhahn J., Bartosch T., Baumbach C.-M., Suchowski M., Kacza J., Schrödl W., Michler J.K. (2021). Detection of subtilisin 3 and 6 in skin biopsies of cattle with clinically manifested bovine ringworm. Med. Mycol..

[47] Jousson O., Léchenne B., Bontems O., Mignon B., Reichard U., Barblan J., Quadroni M., Monod M. (2004). Secreted subtilisin gene family in Trichophyton rubrum. Gene.

[48] Zaugg C., Jousson O., Léchenne B., Staib P., Monod M. (2008). *Trichophyton rubrum* secreted and membrane-associated carboxypeptidases. Int. J. Med. Microbiol..

[49] Burmester A., Shelest E., Glöckner G., Heddergott C., Schindler S., Staib P., Heidel A., Felder M., Petzold A., Szafranski K. (2011). Comparative and functional genomics provide insights into the pathogenicity of dermatophytic fungi. Genome Biol..

[50] Méhul B., Gu Z., Jomard A., Laffet G., Feuilhade M., Monod M. (2016). Sub6 (Tri r 2), an Onychomycosis Marker Revealed by Proteomics Analysis of *Trichophyton rubrum* Secreted Proteins in Patient Nail Samples. J. Investig. Dermatol..

[51] Shi Y., Niu Q., Yu X., Jia X., Wang J., Lin D., Jin Y. (2016). Assessment of the function of SUB6 in the pathogenic dermatophyte *Trichophyton mentagrophytes*. Med. Mycol..

[52] Woodfolk J.A., Wheatley L.M., Piyasena R.V., Benjamin D.C., Platts-Mills T.A.E. (1998). *Trichophyton* Antigens Associated with IgE Antibodies and Delayed Type Hypersensitivity. Sequence homology to two families of serine proteinases. J. Biol. Chem..

[53] Jousson O., Léchenne B., Bontems O., Capoccia S., Mignon B., Barblan J., Quadroni M., Monod M. (2004). Multiplication of an ancestral gene encoding secreted fungalysin preceded species differentiation in the dermatophytes *Trichophyton* and *Microsporum*. Microbiology.

[54] Giddey K., Monod M., Barblan J., Potts A., Waridel P., Zaugg A.C., Quadroni M. (2007). Comprehensive Analysis of Proteins Secreted by *Trichophyton rubrum* and *Trichophyton violaceum* under in Vitro Conditions. J. Proteome Res..

[55] Giddey K., Favre B., Quadroni M., Monod M. (2007). Closely related dermatophyte species produce different patterns of secreted proteins. FEMS Microbiol. Lett..

[56] Preuett B.L., Schuenemann E., Brown J.T., Kovac M.E., Krishnan S.K., Abdel-Rahman S.M. (2010). Comparative analysis of secreted enzymes between the anthropophilic–zoophilic sister species *Trichophyton tonsurans* and *Trichophyton equinum*. Fungal Biol..

