# Supplementary material for: Comprehensive Assessment of the Virulence Factors sub 3, sub 6 and mcpA in the Zoonotic Dermatophyte Trichophyton benhamiae Using FISH and qPCR
Source: J Fungi (Basel). 2021 Dec 28;8(1):24. doi: 10.3390/jof8010024 (PMC8778074; doi:10.3390/jof8010024)
Supplement: Supplementary file 1 [file jof-08-00024-s001.zip › jof-1514255-supplementary.pdf]

Supplementary Materials:

Table S1. Further information on *T. benhamiae* isolates used during this study.

T. benhamiae **isolate**	**source**[a]	**phenotypic appearance** (Sabouraud agar, 15 d, 28 °C)		**ITS type (NCBI acc. no.)**[a]	**mating type [27]**
		**obverse**	**reverse**		
**205848**	hum	white with yellow rim, furrowed	yellow, furrowed		
**205815**	hum	white with yellow rim, furrowed	yellow, furrowed		
**205850**	hum	white with yellow rim, furrowed	yellow, furrowed		
**206139**	hum	white, radial	beige, velvety		
**207002**	hum	white, powdery	beige-brown, velvety		
**207413**	hum	white, powdery	yellow, velvety		
**205858**	hum	white, radial	yellow with white rim	yellow (KU257464.1)	mt -
**206785**	hum	white with yellow rim, furrowed, radial	yellow, furrowed		
**207860**	hum	white, powdery	yellow, velvety		
**206494**	hum	white, radial	yellow with white rim		
**7A**	Gp	white with yellow rim	yellow		
**12A**	Gp	white, powdery	white-beige, velvety		
**15B**	Gp	white with yellow rim	yellow, furrowed		
**1A**	Gp	white with yellow rim	yellow		
**2A**	Gp	white with yellow rim	yellow		
**5A**	Gp	white with yellow rim	yellow		
**14A**	Gp	white with yellow rim	yellow		
**8A**	Gp	white with yellow rim	yellow		
**3A**	Gp	white with yellow rim	yellow		
**6A**	Gp	white with yellow rim (white, radial)	yellow	white (LN874022.1)	

[a] Human-derived (hum) isolates were obtained from patients of the Laboratory of Medical Microbiology (Moelbis, Germany) with informed consent. Isolates of Guinea pig origin (Gp) were collected at a local zoological garden from feed animals as part of a previous project to develop a dermatophyte biobank. All samples were taken according to local ethical guidelines and state law.

Table S2. Name, sequence and further information on the primers used for PCR and qPCR during this study.

**use**	**target** gene	**primer**	**sequence (5´-3´)**	**Tm [C°]**	**amplicon [bp]**	**ref.**
Identification	ITS 1, 5.8 S rRNA and ITS 2	LSU266	GCATTCCCAAACAACTCGACTC	61.5	ca. 1000	[26]
		V9G	TTACGTCCCTGCCCTTTGTA	58.1		
Screening for mt+	HMG partial	MF1	ATGGCCACTACTTCTGGGACC	63.7	307	[27]
		MF2	CATTGGTAAAACAGCAGCCCC	61.5		
Screening for mt-	α-Box partial	MF3	AAAATGTCTGGCACCGAAGTCTC	62.2	380	
		MF4	TCTGGGAGAGATCGGGAAACGC	66.4		
expression rates of virulence factors	*isocitrate lyase*	isol-f	TGGAAAGATTCAAGATGGCGATA	58.6	103	[30]
		isol-rev	TCTGTGCATTTGATGGGTAATCA	58.7		
	subtilisin 3	sub3-f	CATGCTTGCCCAGGGTGTTAG	63.0	81	[5]
		sub3-rev	CCTGGGTTGCGGATAACG	58.7		
	subtilisin 6	sub6-f	GCGGCAGCACTGATACTCTCT	62.7	121	
		sub6-rev	TTGCCCAATCCGATGAGGTA	58.3		
	metallocarbxy peptidase A	mcpA-f	GCATTGAAGGCGGTGCAT	57.9	84	[30]
		mcpA-rev	GTCAACACTGTCTCCATTAACTTGGT	61.4		
Reference gene for normalizatio n	*ADP-ribosylation factor*	ADPrf-f	CAGAGAAGAGTTGCAAAAGATGCT	59.5	74	[30]
		ADPfr-rev	TGCTTGTTGGCGAAAACGA	57.8		

Table S3. The expression rates of the different virulence factors found in human derived *T. benhamiae* isolates were compared (P values; statistically significant differences are defined as P < 0.05 and indicated in bold letters).

Sampling day	human derived *T. benhamiae* isolates					
	sub 3 vs. ***sub 6***	***sub 3*** vs. ***mcpA***	***sub 3*** vs. ***isol***	***sub 6*** vs. ***mcpA***	***sub 6*** vs. ***isol***	***mcpA*** vs. ***isol***
3 (n = 3)	1,000	0,4	1	0,4	0,7	0,7
5 (n = 5)	0,222	0,151	0,151	1	1	0,841
7 (n = 6)	**0,004**	0,065	**0,015**	0,937	0,589	0,937
10 (n = 4)	**0,029**	0,057	**0,029**	**0,029**	**0,019**	**0,029**

Table S4. The transcript numbers of the different virulence factors expressed by Gp derived *T. benhamiae* isolates were compared (P values; statistically significant differences are defined as P < 0.05 and indicated in bold letters).

Sampling day	Gp derived *T. benhamiae* isolates					
	sub 3 vs. ***sub 6***	***sub 3*** vs. ***mcpA***	***sub 3*** vs. ***isol***	***sub 6*** vs. ***mcpA***	***sub 6*** vs. ***isol***	***mcpA*** vs. ***isol***
3 (n = 3)	0,700	0,1	0,1	1	1	1
5 (n = 6)	0,093	0,24	0,31	0,589	0,818	0,937
7 (n = 7)	**<0,001**	**0,017**	**0,007**	**0,002**	0,209	0,165
10 (n = 7)	**<0,001**	**<0,001**	**<0,001**	**<0,001**	0,209	**<0,001**

Table S5. The expression rates of the different virulence factors produced by *T. benhamiae* isolates were compared (P values; statistically significant differences are defined as $P < 0.05$ and indicated in bold letters).

Sampling day	*T. benhamiae* isolates (hum and Gp derived)					
	sub 3 vs. ***sub 6***	***sub 3*** vs. ***mcpA***	***sub 3*** vs. ***isol***	***sub 6*** vs. ***mcpA***	***sub 6*** vs. ***isol***	***mcpA*** vs. ***isol***
3 (n = 6)	0,94	0,18	0,132	0,485	0,937	0,937
5 (n = 11)	**0,02**	**0,049**	**0,049**	0,694	0,743	0,844
7 (n = 13)	**<0,001**	**0,004**	**<0,001**	**0,013**	0,182	0,218
10 (n = 11)	**<0,001**	**<0,001**	**<0,001**	**<0,001**	0,511	**<0,001**
